# Human Wharton’s Jelly Mesenchymal Stem Cell-Mediated Sciatic Nerve Recovery Is Associated with the Upregulation of Regulatory T Cells

**DOI:** 10.3390/ijms21176310

**Published:** 2020-08-31

**Authors:** Aline Yen Ling Wang, Charles Yuen Yung Loh, Hsin-Hsin Shen, Sing-Ying Hsieh, Ing-Kae Wang, Chin-Ming Lee, Chia-Hsien Lin

**Affiliations:** 1 Center for Vascularized Composite Allotransplantation, Chang Gung Memorial Hospital, Taoyuan 333, Taiwan; aimin0703@gmail.com (C.-M.L.); qennel@gmail.com (C.-H.L.); 2 Department of Plastic Surgery, Addenbrooke’s Hospital, Cambridge CB2 0QQ, UK; chloh_yy@hotmail.com; 3 Biomedical Technology and Device Research Laboratories, Industrial Technology Research Institute, Hsinchu 310, Taiwan; shenhsin@itri.org.tw (H.-H.S.); itri990094@itri.org.tw (S.-Y.H.); ikwang@itri.org.tw (I.-K.W.)

**Keywords:** Wharton’s jelly, mesenchymal stem cells, sciatic nerve, transection, neurotmesis, peripheral nerve, regeneration, regulatory T cells, Treg

## Abstract

The acceleration of peripheral nerve regeneration is crucial for functional nerve recovery. Our previous study demonstrated that human Wharton’s jelly-derived mesenchymal stem cells (hWJ-MSC) promote sciatic nerve recovery and regeneration via the direct upregulation and release of neurotrophic factors. However, the immunomodulatory role of hWJ-MSC in sciatic nerve recovery remains unclear. The effects of hWJ-MSC on innate immunity, represented by macrophages, natural killer cells, and dendritic cells, as well as on adaptive immunity, represented by CD4^+^ T, CD8^+^ T, B, and regulatory T cells (Tregs), were examined using flow cytometry. Interestingly, a significantly increased level of Tregs was detected in blood, lymph nodes (LNs), and nerve-infiltrating cells on POD7, 15, 21, and 35. Anti-inflammatory cytokines, such as IL-4 and IL-10, were significantly upregulated in the LNs and nerves of hWJ-MSC-treated mice. Treg depletion neutralized the improved effects of hWJ-MSC on sciatic nerve recovery. In contrast, Treg administration promoted the functional recovery of five-toe spread and gait stance. hWJ-MSC also expressed high levels of the anti-inflammatory cytokines TGF-β and IL-35. This study indicated that hWJ-MSC induce Treg development to modulate the balance between pro- and anti-inflammation at the injured sciatic nerve by secreting higher levels of anti-inflammatory cytokines.

## 1. Introduction

Peripheral nerve injuries differ from central nervous system (CNS) injuries [1], which usually affect end-target organs, thus potentially causing denervation and loss of function. However, peripheral nerve regeneration over a long distance poses a problem. The recovery of movement and sensation are highly time-sensitive processes and the loss of reinnervation [2] can result in the permanent loss of function of a limb, compounding disability, and a decrease in quality of life. Injuries located farther away from their end-target organs or muscles take a much longer time to regenerate, which causes the atrophy of motor end plates, eventually resulting in muscle atrophy [3]. Therefore, a great deal of research has been dedicated to the development of new strategies for accelerating peripheral nerve regeneration to improve the quality of life of patients.

After nerve injury, inflammatory responses play an important role during Wallerian degeneration and axonal growth [4,5,6]. Schwann cells and resident macrophages express inflammatory cytokines, chemokines, and co-stimulatory molecules, which recruit macrophages and lymphocytes from the blood vessels and induce a local inflammatory process. The inflammatory infiltrate is mainly composed of macrophages and is necessary for the removal of myelin debris, which inhibits axonal growth. However, activated macrophages also secrete the pro-inflammatory TNF-α and reactive oxygen species (ROS), which damage nerves. The coordination of pro- and anti-inflammatory signals during Wallerian degeneration is crucial and must be tightly controlled to ensure successful axon regeneration.

Recent interest in mesenchymal stem cells (MSC) and their multipotent potential has led to the expansion of research involving tissue regeneration [7], particularly of the umbilical cord [8,9]. Wharton’s jelly is a gelatinous substance located within the umbilical cord that is largely made up of mucopolysaccharides, such as hyaluronic acid and chondroitin sulfate. MSC can be isolated from Wharton’s jelly from the umbilical cord, which contains mucoid connective tissue and fibroblast-like cells. Our previous study demonstrated that the topical application of human Wharton’s jelly-derived MSC (hWJ-MSC) promote sciatic nerve recovery and regeneration because of the direct upregulation and release of neurotrophic factors [10]. MSC also exhibit diverse immunomodulatory effects, in addition to their regenerative property [11,12]. However, it remains unclear whether hWJ-MSC have the potential immunologically to regulate peripheral nerve regeneration. Studies indicate that the inhibition of inflammatory cell recruitment and activation decreases inflammatory immune responses and improves peripheral nerve regeneration [13,14,15,16,17]. Interestingly, in vaccination models for the treatment of CNS injury, Th2-inducing adjuvants, such as IFA and Alum, promote axonal regeneration better than does the Th1-inducing adjuvant CFA [18,19]. Th2 cells can inhibit pro-inflammatory Th1 and Th17 cells via the secretion of anti-inflammatory cytokines, such as IL-4 and IL-10. Therefore, we hypothesized that hWJ-MSC possess anti-inflammatory properties for the acceleration of sciatic nerve recovery and regeneration. 

Regulatory T (Treg) cells exhibit anti-inflammatory properties via four main mechanisms [20,21,22,23]. Treg cells secrete cytolytic enzymes, such as granzyme A and B, which can destroy effector T cells. Treg cells are also able to soak up IL-2 cytokines in the microenvironment, which eliminates this crucial cytokine that is required for inflammatory cell survival. They also exert their effects via cell-to-cell contact with dendritic cells (DCs), thus inhibiting their function by blocking co-stimulatory molecules (CD80 and CD86) with CTLA4. Studies demonstrated that CD4^+^CD25^+^ Treg cells have neuroprotective activities, which promote nerve survival [24]. Activated T cells can secrete the brain-derived neurotrophic factor (BDNF), which can promote nerve regeneration [25]. Therefore, we assumed that hWJ-MSC-mediated sciatic nerve recovery and regeneration occur partly via the upregulation of Treg cells and provides a balance between pro- and anti-inflammatory signals during Wallerian degeneration.

## 2. Results

### 2.1. Effects of hWJ-MSC on Innate Immunity in Sciatic Nerve-Injured Mice

**Figure 1:**
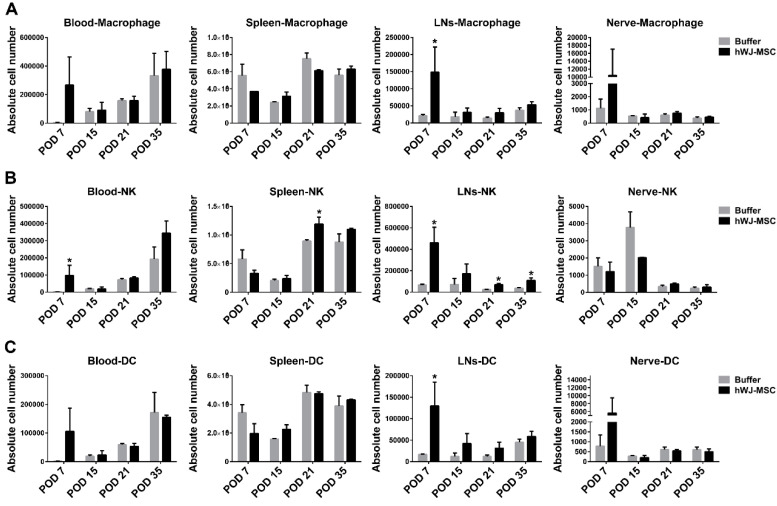
Effects of human Wharton’s jelly-derived mesenchymal stem cells (hWJ-MSC) on innate immunity in sciatic nerve-injured mice. (**A**–**C**) The absolute number of macrophages, natural killer cells (NKs), and dendritic cells (DCs) in blood, spleen, lymph nodes (LNs), and nerve-infiltrating cells. Immune cells in the two groups (*n* = 3) were harvested and analyzed using flow cytometry on POD7, 15, 21, and 35. The cells were stained to identify CD11b^+^ macrophages, NK1.1^+^ NKs, and CD11c^+^ DCs. The statistical data were represented as the mean ± SD. * *p* < 0.05.

Our previous study demonstrated that the topical application of hWJ-MSC accelerates signs of sciatic nerve recovery, such as five-toe spread and toe-off phase, partly via the direct upregulation and release of neurotrophic factors (Supplementary Figure S1A,B) [10]. To investigate the immunomodulatory effects of hWJ-MSC on sciatic nerve recovery, various immune cell populations were analyzed after hWJ-MSC administration to the nerve coaptation site. Lymphoid organs, such as blood, spleen, and lymph nodes (LNs) near the anastomosed nerve, as well as immune cells infiltrated within the anastomosed nerve, were harvested and analyzed using flow cytometry on postoperative days (POD) 7, 15, 21, and 35. Innate immunity was represented by macrophages, natural killer cells (NKs), and dendritic cells (DCs). Macrophages and DCs were detected at a similar level in lymphoid organs and nerve-infiltrating cells between the buffer and hWJ-MSC groups at four time points (but not at POD7) (Figure 1). NKs exhibited a similar level in blood, spleen, and nerve-infiltrating cells over time. In contrast, NKs displayed a trend toward a significant increase in LNs over time, possibly because of the increase in total cell number in LNs (Supplementary Figure S2). Interestingly, the total number of nerve-infiltrating cells displayed a decreasing trend, suggesting that the inflammatory immune response may be suppressed in hWJ-MSC-treated mice. Blood testing revealed a similar total number of lymphocytes, monocytes, and granulocytes between the two groups (Supplementary Figure S3). These results suggest that hWJ-MSC have no significant effect on macrophages, NKs, and DCs over time, except for LNs and POD7.

### 2.2. Effects of hWJ-MSC on Adaptive Immunity in Sciatic Nerve-Injured Mice

**Figure 2:**
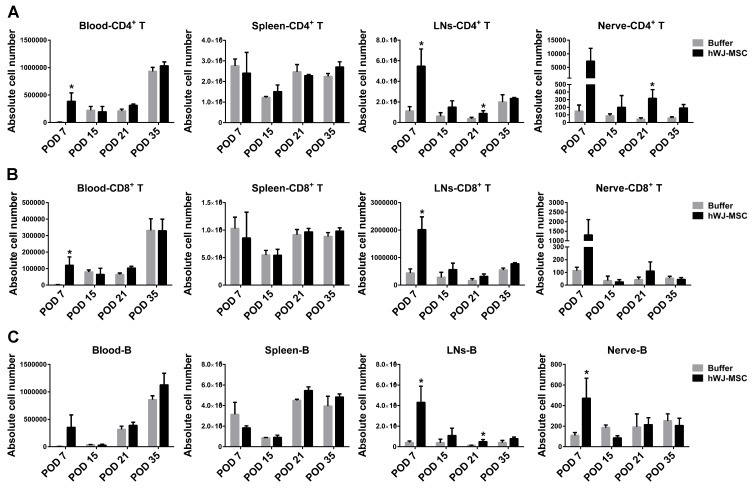
Effects of hWJ-MSC on adaptive immunity in sciatic nerve-injured mice. (**A**–**C**) The absolute number of CD4^+^ T, CD8^+^ T, and B cells in blood, spleen, LNs, and nerve-infiltrating cells. Immune cells in the two groups (*n* = 3) were harvested and analyzed using flow cytometry on POD7, 15, 21, and 35. The cells were stained to identify CD4^+^ T, CD8^+^ T, and CD19^+^ B cells. The statistical data were represented as the mean ± SD. * *p* < 0.05.

To investigate the effects of hWJ-MSC on adaptive immunity, T and B cells were analyzed. T cells are a population of lymphocytes that express a T cell receptor on their cell surface. Antigen-presenting cells, such as DCs, present antigens to T cell receptors to stimulate T cell activation. B cells are also a population of lymphocytes, that are responsible for humoral immunity via the secretion of antibodies. CD4^+^ T cells were detected at similar levels in blood and spleen. However, an increasing trend was observed in LNs and nerve-infiltrating cells over time (Figure 2). CD8^+^ T and B cells exhibited similar levels in lymphoid organs and nerve-infiltrating cells between the two groups, except for LNs and blood at POD7 which demonstrated an increase in the hWJ-MSC group.

### 2.3. hWJ-MSC Upregulate Treg Cells in Lymphoid Organs and Nerve-Infiltrating Cells over Time

To further evaluate the balance between pro-inflammation and anti-inflammation in hWJ-MSC-treated mice, we used a Luminex multiplex assay to detect the expression of cytokine proteins, such as the pro-inflammatory TNF-α, IL1β, IL-2, IFN-γ, and IL17A and the anti-inflammatory IL-4 and IL-10. These cytokines were not detected in serum samples, in addition to the similarly low levels of TNF-α and IL-17A (around 1–5 pg/mL) observed between the buffer and hWJ-MSC groups. The pro-inflammatory TNF-α, IL1β, IL-2, IFN-γ, and IL17A cytokines appeared to be decreased in the LNs of hWJ-MSC-treated mice. In particular, a significantly decreased level of IL-2 was detected on POD7. Moreover, a significantly decreased level of TNF-α and IL-2 was observed in the anastomosed nerve over time (Supplementary Figure S4). Interestingly, anti-inflammatory cytokines, such as IL-4 and IL-10, were significantly upregulated in the LNs and nerve of hWJ-MSC-treated mice over time (Supplementary Figure S5).

**Figure 3:**
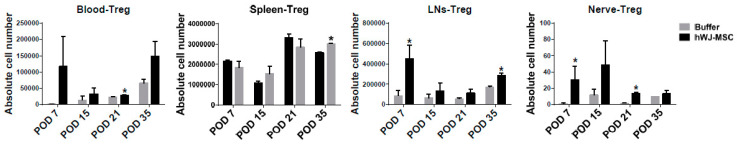
Effects of hWJ-MSC on Treg cells in sciatic nerve-injured mice. The absolute number of regulatory T cells (Tregs) in blood, spleen, LNs, and nerve-infiltrating cells. Tregs in the two groups (*n* = 3) were harvested and analyzed using flow cytometry on POD7, 15, 21, and 35. The cells were stained to identify CD4^+^CD25^+^FoxP3^+^ Tregs. The statistical data were represented as a mean ± SD. * *p* < 0.05.

The results reported above show that immunomodulation occurred in hWJ-MSC-treated mice, suggests that anti-inflammatory T cells may be induced to suppress the inflammation elicited by the injured nerve. To confirm this hypothesis, we examined the CD4^+^CD25^+^Foxp3^+^ Treg cell population in lymphoid organs and nerve-infiltrating cells over time. Treg cells, which are crucial for anti-inflammation because of anti-inflammatory cytokine secretion, were significantly increased in LNs and nerve-infiltrating cells (Figure 3). The absolute number (mean ± SD) of Tregs in the blood, spleen, LNs, and nerve-infiltrating cells over time between the buffer and hWJ-MSC groups are shown in Supplementary Figure S6. The spleen also displayed a significant increase in Tregs on POD35. These results suggest that hWJ-MSC significantly induce Treg development and recruit Tregs into the injured sciatic nerve, to achieve a balance between pro- and anti-inflammation.

### 2.4. Treg Depletion Neutralizes the Benefical Effects of hWJ-MSC on Sciatic Nerve Recovery

**Figure 4:**
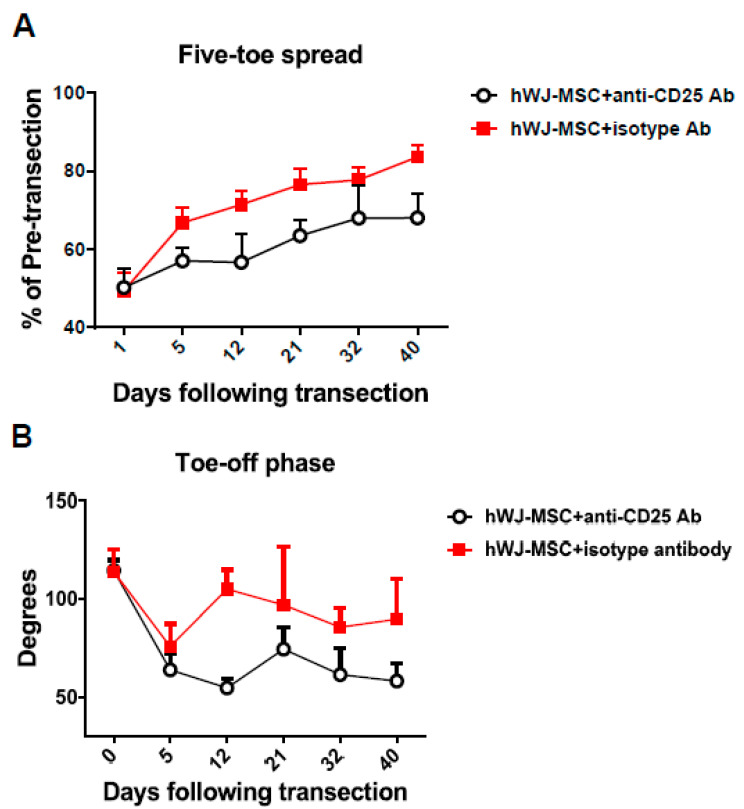
Anti-CD25 antibody administration eliminated the hWJ-MSC-mediated functional recovery in hindlimbs. (**A**) An anti-CD25 antibody worsened the functional recovery of the five-toe spread reflex. hWJ-MSC (5 × 10^5^) in 50 μL were added to the space created in the musculature of each mouse. Progressive chart of the percentage of the pre-transection five-toe spread distance measured over time after the division of the sciatic nerve in the anti-CD25- and isotype-treated groups. The differences between the mean ± SD of the two groups were significant (*p* value < 0.05, one-way analysis of variance (ANOVA); anti-CD25 vs. isotype control, *p* = 0.01; Tukey’s test). The average five-toe spread in the normal mouse group was 9.811 ± 0.057 mm. (**B**) The anti-CD25 antibody worsened gait stance and movement. The angles were measured in both groups after transection surgery. The mean ± SD values were significantly different in the two groups (*p* value < 0.05, one-way ANOVA; anti-CD25 vs. isotype control, *p* = 0.04; Tukey’s test). The average angle of the normal mouse group was 110.0 ± 0.72.

To verify whether hWJ-MSC-induced Tregs contribute to the functional recovery of the injured sciatic nerve, an anti-CD25 antibody was administrated to hWJ-MSC-treated mice for the depletion of Tregs. Functional recovery was identified based on five-toe spread and video gait analyses. Each five-toe spread distance measurement was expressed as a percentage of the pre-transection distance, to monitor recovery, with a higher percentage indicating a better recovery. On the other hand, the ankle angle was plotted against time, with a greater angle indicating a better gait stance and movement. We found that both the five-toe spread and gait stance curves of the anti-CD25 antibody treatment group were significantly decreased compared with the isotype control antibody group of hWJ-MSC-treated mice (Figure 4A,B). These results indicate that the depletion of Tregs eliminates the beneficial effects of hWJ-MSC on the functional recovery of five-toe spread and gait movement. Tregs may participate in the functional recovery and regeneration of the injured sciatic nerve.

### 2.5. Tregs Promote the Functional Recovery of the Sciatic Nerve

**Figure 5:**
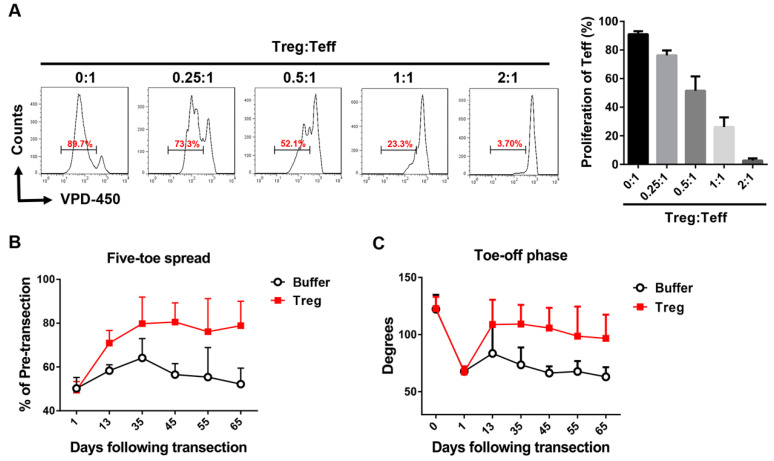
Treg administration improved the functional recovery of the sciatic nerve. (**A**) CD4^+^CD25^+^ Tregs (Treg) were isolated with a purity of >90% from naïve mice using a mouse CD4^+^CD25^+^ regulatory T cell isolation kit and an autoMACS instrument. CD4^+^CD25^−^ effector T cells (Teff) were isolated with a purity of >90% using an autoMACS instrument. Tregs were co-cultured with VPD-450-labeled effector T cells under anti-CD3 (2 μg/mL) and anti-CD28 (2 μg/mL) stimulation for 3 days before analysis using flow cytometry. The difference between the mean ± SD values of the Treg mixture (0.25~2:1) and Teff (0:1) groups was significant in all cases (*p* < 0.005, Student’s *t*-test). (**B**) Tregs promoted the functional recovery of the five-toe spread reflex. Isolated Tregs (5 × 10^5^) with a purity of >90% were added to the space created in the musculature of each mouse. Progressive chart of the percentage of the pre-transection five-toe spread distance measured over time after the division of the sciatic nerve in the buffer and Treg groups. The differences between the mean ± SD values of the two groups were significant (*p* value < 0.05, one-way ANOVA; Treg vs. buffer control, *p* = 0.02; Tukey’s test). The average five-toe spread in the normal mouse group was 9.950 ± 0.158 mm. (**C**) Tregs promoted gait-stance and movement improvement. The angles were measured in both groups after the transection surgery. Comparison of the mean ± SD values in the two groups revealed the presence of a significant difference (*p* value < 0.005, one-way ANOVA; Treg vs. buffer control, *p* = 0.01; Tukey’s test). The average angle of the normal mouse group was 122.5 ± 2.89.

To confirm further whether Tregs contribute to the functional recovery of the sciatic nerve, purified naïve CD4^+^CD25^+^ Tregs were administrated to the injured sciatic nerve. Functional recovery was identified based on five-toe spread and video gait analyses. Purified Tregs afforded good immunosuppression regarding effector T cell proliferation in a dose-dependent manner (Figure 5A). Both the five-toe spread and gait stance curves of the Treg group were significantly improved compared with the buffer group (Figure 5B,C). These results indicate that hWJ-MSC administration accelerates the functional recovery of the injured sciatic nerve and is associated with Treg upregulation.

2.6. hWJ-MSC Express High Levels of TGF-β and IL-35

**Figure 6:**
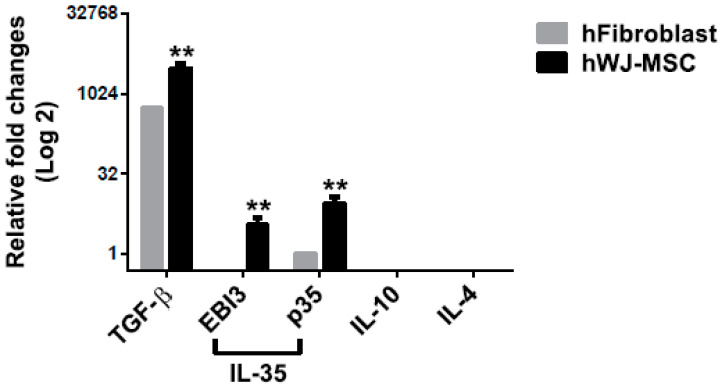
Quantification of hWJ-MSC-derived cytokine expression. The expression of mRNAs for Treg-associated cytokines, such as TGF-β, IL-10, IL-4, and IL-35 (which are composed of EBI3 and p35), in hWJ-MSC and fibroblasts, was quantified using qPCR. The levels of expression of cytokines were was normalized to that of GAPDH. The difference between the mean ± SD values of the hWJ-MSC and fibroblast groups was significant (** *p* < 0.005, Student’s *t*-test).

Increased Treg cell development may be possible via the upregulation of cytokines for Treg cell differentiation from hWJ-MSC. To validate this possibility, Treg-associated cytokines, such as TGF-β, IL-10, IL-4, and IL-35 composed of EBI3 and p35, were analyzed in hWJ-MSC and control human fibroblasts. TGF-β and IL-35 can induce Treg cell differentiation, and Tregs secrete the TGF-β, IL-10, IL-4, and IL-35 cytokines for anti-inflammation. hWJ-MSC expressed higher levels of TGF-β and IL-35 compared with fibroblasts (Figure 6). These results suggest that hWJ-MSC promote sciatic nerve recovery and regeneration and are partly associated with increased Treg-associated cytokines, in addition to the upregulation of neurotrophic factors. Increased Treg cells contribute to the coordination of pro- and anti-inflammation during sciatic nerve regeneration.

## 3. Discussion

This study investigated the immunomodulatory effects of hWJ-MSC on nerve regeneration in a mouse model of simple transection mimicking that commonly encountered in clinical practice. To our knowledge, this study was the first to examine the immunomodulatory possibility of using hWJ-MSC to enhance peripheral nerve regeneration in a mouse model of complete transection and repair of the sciatic nerve. Increased Treg cells were found in the blood, spleen, LNs, and nerve-infiltrating cells of hWJ-MSC-treated mice on POD7, 15, 21, and 35, as assessed by flow cytometry. Increased anti-inflammatory cytokines, such as IL-4 and IL-10, were also detected in hWJ-MSC-treated mice over time by Luminex multiplex assay. The pro-inflammatory TNF-α, IL1β, IL-2, IFN-γ, and IL17A cytokines showed a decreasing trend in the LNs of hWJ-MSC-treated mice. Decreased levels of TNF-α, IL1β, and IL-2 were also observed in the nerve tissues of hWJ-MSC-treated mice. These results suggest that hWJ-MSC may induce anti-inflammation to control pro-inflammation in LNs and the sciatic nerve. Moreover, Treg cell depletion eliminated the hWJ-MSC-mediated sciatic nerve recovery, whereas Treg cell administration improved the functional recovery of five-toe spread and gait stance. hWJ-MSC also exhibited high levels of expression of the anti-inflammatory TGF-β and IL-35 cytokines. These results suggest that hWJ-MSC promote sciatic nerve recovery and regeneration and are partly associated with the upregulation of Treg-associated cytokines, in addition to the upregulation of neurotrophic factors. Increased Treg cells contribute to the coordination of pro- and anti-inflammation during peripheral nerve regeneration.

These results clarified the immunomodulatory mechanism of hWJ-MSC. These cells secrete higher levels of anti-inflammatory cytokines, such as IL-35 and TGF-β, for the direct suppression of inflammation in the injured peripheral nerve. hWJ-MSC also upregulate Treg cell development by secreting TGF-β and IL-35, which can induce Treg cell differentiation. Subsequently, increased Treg cells are recruited into the injured peripheral nerve from lymphoid organs and modulate nerve pro-inflammation. Although activated macrophages remove myelin debris for axonal regeneration, they also secrete pro-inflammatory TNF-α and ROS, which adversely affect nerve regeneration. The balance between pro- and anti-inflammation is crucial for peripheral nerve regeneration. Thus, hWJ-MSC-induced anti-inflammatory immune responses are important for controlling inflammation in the damaged sciatic nerve. This study provided a basis for neuroregenerative medicine by elucidating the mechanisms underlying the anti-inflammatory immunomodulation in the sciatic nerve functional recovery and further inspire new strategies of nerve regeneration via the use of anti-inflammatory drugs.

The effects of hWJ-MSC on innate immunity (represented by macrophages, NKs, and DCs) and on adaptive immunity (represented by CD4^+^ T, CD8^+^ T, and B cells) were examined using flow cytometry. These immune cells showed a similar level between the buffer and hWJ-MSC groups, except for LNs and POD7. The hWJ-MSC group showed an increasing trend of these immune cells in LNs, as well as an increasing trend in blood, LNs, and nerve-infiltrating cells on POD7 (Figure 1 and Figure 2). Although these immune cell populations were increased in such tissues of hWJ-MSC-treated mice, pro-inflammatory cytokines were not upregulated in these animals. Moreover, the anti-inflammatory IL-4 and IL-10 cytokines were significantly increased in such tissues of hWJ-MSC-treated mice. Therefore, we propose that these immune cells control pro-inflammation in injured sciatic nerves by secreting anti-inflammatory cytokines. In addition to CD4^+^CD25^+^ Tregs, which secrete anti-inflammatory cytokines, CD8^+^ T cells, B cells, macrophages, NKs, and DCs can also be classified into subpopulation, such as CD4^+^FoxP3^−^ type 1 regulatory T cells (Tr1) [26], regulatory CD8^+^ T cells [27], regulatory B (Breg) cells [28], M2 macrophages [29], regulatory NKs [30], and regulatory DCs [31], which specifically release anti-inflammatory cytokines and modulate pro-inflammation. Previous evidence showed that IL-35 induces the differentiation and development of Tregs and Bregs [32]. MSC induce regulatory lymphocytes through the secretion of multiple pleiotropic cytokines and cell-to-cell contact with target cells [33,34]. On the other hand, CD4^+^ T cell subsets, such as Th1, Th17, Th2, Tr1, and Treg cells, showed a peak expansion and cytokine production on POD7, as assessed using flow cytometry analysis [35], which is similar to the results reported here (Figure 1 and Figure 2, Supplementary Figures S4 and S5). Regulatory immune cells may be activated and recruited during the first week after sciatic nerve injury and Wallerian degeneration. Therefore, hWJ-MSC may induce these regulatory immune cells via the secretion of high levels of TGF-β and IL-35, to modulate the balance between pro- and anti-inflammation, for the subsequent improvement of sciatic nerve recovery.

Previous studies have demonstrated that Th2-inducing adjuvants accelerate axonal regeneration better than does the Th1-inducing adjuvant CFA [18,19] in vaccination models of the treatment of CNS injury. Th2 cells suppress pro-inflammation through the secretion of the anti-inflammatory IL-4 and IL-10 cytokines, which is similar to that reported here (Supplementary Figures S4 and S5). hWJ-MSC also upregulate IL-4 and IL-10 to control pro-inflammation for the improvement of sciatic nerve recovery. Previous evidence indicated that simvastatin improves the morphological and functional recovery of sciatic nerve injury in Wistar rats via interference with innate and acquired immunity [14]. Simvastatin-treated rats showed a decreased level of mononuclear cell infiltration during Wallerian degeneration and nerve regeneration on POD7, 14, and 21, which is similar to our results (Supplementary Figure S2). Simvastatin induces Th2 cells, reduces the expression of pro-inflammatory cells, and promotes Treg cell activation in experimental autoimmune encephalitis [36]. Our results were also similar to those of a study that reported the upregulation of the Th2-associated IL-4 and IL-10 cytokines in hWJ-MSC-treated mice (Supplementary Figure S5). The literature demonstrated that the adoptive transfer of CD4^+^CD25^+^ Tregs yielded neuroprotective effects through the suppression of microglial responses to stimuli in an animal model of Parkinson’s disease [24]. A Treg population expander, CD28SupA, significantly attenuated the neuropathic pain resulting from sciatic nerve injury, whereas the depletion of Tregs by a CD25 antibody in a model with partial sciatic nerve ligation resulted in prolonged mechanical pain hypersensitivity [37]. These findings, together with our results, suggest that Tregs play a critical role in neuroregeneration, neuroprotection, and recovery from neuropathy-induced pain.

hWJ-MSC have been shown to be negative for major histocompatibility complex class (MHC) II and exhibit low expression of MHC class I molecules [38,39]. Their low-immunogenicity property ensures the survival of hWJ-MSC in allogeneic hosts. Therefore, their low immunogenicity renders them ideal candidates for use in cell therapeutics and adequate for use as an allogeneic cell bank in the future.

## 4. Materials and Methods

### 4.1. Animals

The 6–8 week old of BALB/c mice were purchased from the National Laboratory Animal Center, Taiwan. All mouse procedures carried out in the study were fully compliant with the recommendations stipulated by the Guide for the Care and Use of Laboratory Animals of Chang Gung Memorial Hospital Animal Research Guidelines. Mouse protocols were approved by the Committee on the Ethics of Animal Experiments of the Chang Gung Memorial Hospital (CGMH) in Taiwan and Institutional Animal Care and Use Committees (IACUC) of CGMH in Taiwan under permit numbers IACUC 2018080701 (approval date: 5 September 2018), and IACUC 2019010201 (approval date: 13 March 2019). The mice were cared for in an enriched environment with an abundance of nesting material. No adverse effects were found in our mouse experiments.

### 4.2. Harvest and Culture of hWJ-MSC

hWJ-MSC harvest was approved by the Ethics Committee of CGMH and Industrial Technology Research Institute (ITRI) under permit number 201700728A3C601 (approval date: 12 December 2017). Dr. Hsin-Hsin Shen from the ITRI provided hWJ-MSC isolated from the Wharton’s jelly of the human umbilical cord. The umbilical cord was obtained from a healthy donor who provided prior informed written consent to the CGMH. The determination of donor eligibility was performed according to the Donor Eligibility Determination Guidelines for Human Cell Therapy Products. The umbilical cord was washed extensively in phosphate-buffered saline (PBS, Corning, NY, USA). Wharton’s jelly tissues were then stripped from the umbilical cord using forceps and scalpels, cut into small pieces with scissors, and digested in 2 mg/mL collagenase NB 4 Standard Grade (SERVA Electrophoresis GmbH, Heidelberg, Germany) for 16–20 h at 37 °C, to release hWJ-MSC. hWJ-MSC were then collected by centrifugation at 1500 rpm for 15 min and cultured in SF1 hMSC medium (serum-free medium, Unimed Healthcare Inc., Taipei, Taiwan). The medium was changed every 3 days. When the hWJ-MSC had proliferated to 80% confluence, they were subcultured for a few passages and used as cell sources in the subsequent experiments.

### 4.3. Treg Suppression Assay

The Treg suppression assay was described in our previous study [23]. CD4^+^CD25^+^ Tregs (mouse CD4^+^CD25^+^ Regulatory T Cell Isolation Kit, Miltenyi Biotec #130-091-041, Bergisch Gladbach, Germany) were purified (>90%) from naïve lymphocytes using an autoMACS Pro Separator (Miltenyi Biotec, Bergisch Gladbach, Germany). CD4^+^CD25^−^ effector T cells were isolated with a purity of >90% from wild-type mice using an autoMACS Pro Separator. Treg cells were then co-cultured with VPD-450-labeled effector T cells under anti-CD3 (2 μg/mL) and anti-CD28 (2 μg/mL) stimulation for 3 days before analysis using flow cytometry.

### 4.4. Mouse Sciatic Nerve Transection Model

The mouse sciatic nerve was surgically divided as described previously [40,41]. Mouse anesthesia was maintained with inhaled isoflurane. The posterior surface of the right hindlimb was shaved and cleaned with 75% alcohol. The right sciatic nerve was then exposed via dissection of the gluteal musculature along a fascial plane. The sciatic nerve was transected 1 cm proximal to the trifurcation of the nerve. Epineural repair of the nerve was performed under an operative microscope with 25× magnification using four evenly spaced 10-0 nylon sutures.

### 4.5. Topical Application of hWJ-MSC, Tregs and Buffer Control

After epineural repair of the sciatic nerve, a suspension of cells or buffer was added to the pocket created from the separation of gluteal muscles, immersing the transected sciatic nerve in the cells. The cells (5 × 10^5^) were administrated in each mouse with the same volume (50 μL). The buffer control group had PBS added alone without any cells. The skin was then sutured closed and restored to its appropriate anatomical.

### 4.6. Flow Cytometry Analysis

Immune cells were stained with antibodies for cell surface and intracellular staining. Antibodies such as CD4, CD8, CD19, CD11c, CD11b, NK1.1, CD25, FoxP3 for flow cytometry were purchased from eBioscience (San Diego, CA, USA) and BD Biosciences. Characteristic markers were used to identify each lymphocyte type, as follows: anti-CD4-APC for CD4^+^ T cells, anti-CD8-PE for CD8^+^ T cells, anti-CD19-APC for B cells, anti-CD11c-APC for DCs, anti-CD11b-PE for macrophages, and anti-NK1.1-PE-Cy7 for NKs. These markers are expressed on the immune cell surface; thus, they were identified by cell surface staining. Immune cells were stained with antibodies against these cell surface markers for 30 min at 4 °C and washed for analysis on a FACSCanto II flow cytometer (BD Biosciences, San Jose, CA, USA). An intracellular staining technique was performed using the following stains: Tregs were identified using CD4^+^CD25^+^FoxP3^+^ markers because of the expression of FoxP3 in the cell nucleus. Briefly, immune cells were stained with anti-CD4-APC and anti-CD25-PE for 30 min at 4 °C and washed for fixation/permeabilization (eBioscience, San Diego, CA, USA) for 30 min at 4 °C. Finally, cells were stained with anti-FoxP3-PerCP for 30 min. For the analysis of Tregs, live cells were gated first, followed by gating of CD4^+^ T cells, then CD25^+^FoxP3^+^-expressing cells.

### 4.7. Cytokine analysis by ProcartaPlex Immunoassay

Blood serum, LNs, and anastomosed sciatic nerves of the buffer and hWJ-MSC groups were harvested on POD7 and 15. RIPA buffer was added to the shredded tissues, such as sciatic nerves (100 mg) and nearby LNs, for ultrasonication, followed by centrifugation at 16,000× *g* for 10 min. The supernatants were collected for the measurement of cytokines, such as TNF-α, IL1β, IL-2, IFN-γ, IL17A, IL-4, and IL-10 using a ProcartaPlex multiplex assay kit (Thermo Fisher Scientific, Waltham, MA, USA) on a Luminex instrument platform.

### 4.8. Isolation of Nerve-Infiltrating Cells

The anastomosed sciatic nerve and adjacent muscle (around 100 mg) were harvested on POD7, 15, 21, and 35. Digestion buffer consisting of 0.8 mg/mL collagenase type 4 (Worthington, Lakewood, NJ, USA) and 10% FBS in HBSS was added to the shredded tissues. The mixtures were incubated for 1 h and washed with buffer (2% FBS in HBSS), followed by removal of the debris using a 100 μm filter. Nerve-infiltrating cells were isolated via Ficoll gradient centrifugation and their composition was analyzed using flow cytometry.

### 4.9. Treg Depletion

PC61 anti-CD25 and G0114F7 isotype control antibodies were purchased from BioLegend (San Diego, CA, USA). For Treg depletion, mice were administered with 500 μg of PC61 or G0114F7 antibody in PBS intraperitoneal injection at days -3 and 3 post-operation.

### 4.10. Functional Recovery Analysis Based on Five-Toe Spread

The recovery of the five-toe reflex of mice after nerve transection is a sensitive indicator of intrinsic muscle recovery [42,43]. The distance between the first and last toes was measured using calibrated calipers. The normal five-toe spread distance was measured before transection of the right sciatic nerve. The distance of the five-toe spread was persistently measured at different time points after transection of the right sciatic nerve. Each experimental mouse was measured in four replicate recordings and the average score was used. The five-toe spread distance was expressed as a percentage of the pre-transection distance, to monitor the functional recovery of the right sciatic nerve. The distance was plotted against time, with a greater toe-spread distance indicating a better nerve recovery.

### 4.11. Functional Recovery Analysis Based on Video Gait Angle

Video gait analysis is a noninvasive test that most accurately reflects and correlates with the quantitative measurement of isometric tetanic muscle force, which is also used to evaluate the functional recovery of the sciatic nerve. Sciatic nerve regeneration affects the innervation and maintenance of the muscle bulk that permits normal gait. In particular, the sciatic nerve innervates a large volume of muscle that controls movement at the ankle level. Therefore, the ankle movement of the mouse during walking is affected after nerve transection. The mouse gait cycle comprises four main stages: the foot on the ground, midstance, toe-off, and mid-swing phases. At the toe-off phase, the ankle angle created is indicative of the isometric force generated to lift the mouse’s body weight off the floor [44]. The angle measured during the toe-off phase is correlated with muscle strength and sciatic nerve recovery.

A walking track apparatus was used to guide the mice and a 60 Hz digital image camera was used to record the gait motion. The recording was then repeated four times during walking; the ankle angles were measured for each attempt and the average was calculated.

### 4.12. Real-Time Polymerase Chain Reaction (qPCR)

hWJ-MSC and human fibroblasts (CCD-966SK) were cultured and their mRNAs were extracted and converted to complementary DNA (cDNA). The mRNA expression was analyzed with TaqMan gene expression assays (Thermo Fisher Scientific, Waltham, MA, USA). To examine Treg-associated cytokines, such as human Interleukin-10 (IL-10: Hs00961622_m1), human transforming growth factor (TGF-β: Hs0098133_m1), human Interleukin-4 (IL-4: Hs00174122_m1), Epstein-Barr Virus-Induced 3 (EBI3: Hs00194957_m1), and p35 (p35: Hs01073447_m1), their mRNA levels were quantified using qPCR. The cytokine expression in this study was normalized to that of glyceraldehyde 3-phosphate dehydrogenase (GAPDH: Hs99999905_m1).

### 4.13. Statistical Analysis

All data were expressed as the mean ± SD. The statistical significance of the five-toe spread and gait analysis was evaluated using a one-way analysis of variance (ANOVA) with post hoc analysis by Tukey’s multiple comparison test. The significance between groups was calculated using the two-tailed Student’s *t*-test. All calculations were conducted using GraphPad Prism 6 software (GraphPad Software, San Diego, CA, USA). *p* < 0.05 was considered to be statistically significant.

## 5. Conclusions

This study demonstrated that hWJ-MSC secrete higher levels of anti-inflammatory cytokines, such as TGF-β and IL-35, to modulate the balance between pro- and anti-inflammation by upregulating Treg cell development. Treg depletion using an anti-CD25 antibody neutralized the beneficial effects of hWJ-MSC on sciatic nerve recovery, which was evaluated based on five-toe spread and gait stance analyses. This study provides valuable information on the immunomodulatory effects of hWJ-MSC, thus improving our understanding of the potential application of anti-inflammatory strategies in neuroregenerative and neuroprotective medicine.

## Supplementary Materials

Supplementary materials can be found at https://www.mdpi.com/1422-0067/21/17/6310/s1. Supplementary Figure S1. hWJ-MSC promoted the functional recovery of injured and repaired sciatic nerves in the extremities. (A) hWJ-MSC accelerated the functional recovery of the five-toe spread reflex. Progressive chart of the percentage of the pre-transection five-toe spread distance measured over time after the division of the sciatic nerve in the buffer and hWJ-MSC groups. The differences between the mean ± SD of the buffer and hWJ-MSC groups were significant (*p* value < 0.0001, one-way ANOVA; buffer vs. hWJ-MSC, *p* = 0.0001; Tukey’s test). The average five-toe spread in the normal mouse group was 9.911 ± 0.04832 mm. (B) hWJ-MSC restored normal gait stance and movement. The angles were measured in the buffer and hWJ-MSC groups for up to 293 days after the transection surgery. Comparison of the mean ± SD values in the two groups revealed the presence of a significant difference (*p* = 0.0008, one-way ANOVA; buffer vs. hWJ-MSC, *p* = 0.0030; Tukey’s test). The average angle of the normal mouse group was 128.0° ± 0.8376°. Supplementary Figure S2. Total absolute number of immune cells in blood, spleen, LNs, and nerve-infiltrating cells between the buffer and hWJ-MSC groups over time. Immune cells in the two groups (*n* = 3) were harvested and analyzed using flow cytometry on POD7, 15, 21, and 35. The statistical data were represented as the mean ± SD. * *p* < 0.05. Supplementary Figure S3. The absolute number of lymphocytes, monocytes, and granulocytes in blood between the buffer and hWJ-MSC groups over time. Blood cells in the two groups (*n* = 3) were harvested and analysed using flow cytometry on POD7, 15, 21, and 35. The statistical data were represented as the mean ± SD. * *p* < 0.05. Supplementary Figure S4. Pro-inflammatory cytokine expression. Blood serum, LNs, and anastomosed sciatic nerves in the buffer and hWJ-MSC groups (*n* = 3) were harvested on POD7 and 15. The cytokine expression of TNF-a, IL1b, IL-2, IFN-g, and IL17A was analyzed using ProcartaPlex Immunoassays and Luminex instrument platform. The statistical data were represented as the mean ± SD. * *p* < 0.05. Supplementary Figure S5. Anti-inflammatory cytokine expression. Blood serum, LNs, and anastomosed sciatic nerves in the buffer and hWJ-MSC groups (*n* = 3) were harvested on POD7 and 15. The cytokine expression of IL-4 and IL-10 was analyzed using ProcartaPlex Immunoassays and Luminex instrument platform. The statistical data were represented as the mean ± SD. * *p* < 0.05. Supplementary Figure S6. The absolute number (mean ± SD) of Tregs in blood, spleen, LNs, and nerve-infiltrating cells over time between the buffer and hWJ-MSC groups.
