# Supplementary material for: Human Wharton’s Jelly Mesenchymal Stem Cell-Mediated Sciatic Nerve Recovery Is Associated with the Upregulation of Regulatory T Cells
Source: Int J Mol Sci. 2020 Aug 31;21(17):6310. doi: 10.3390/ijms21176310 (PMC7504196; doi:10.3390/ijms21176310)
Supplement: Supplementary file 1 [file ijms-21-06310-s001.pdf]

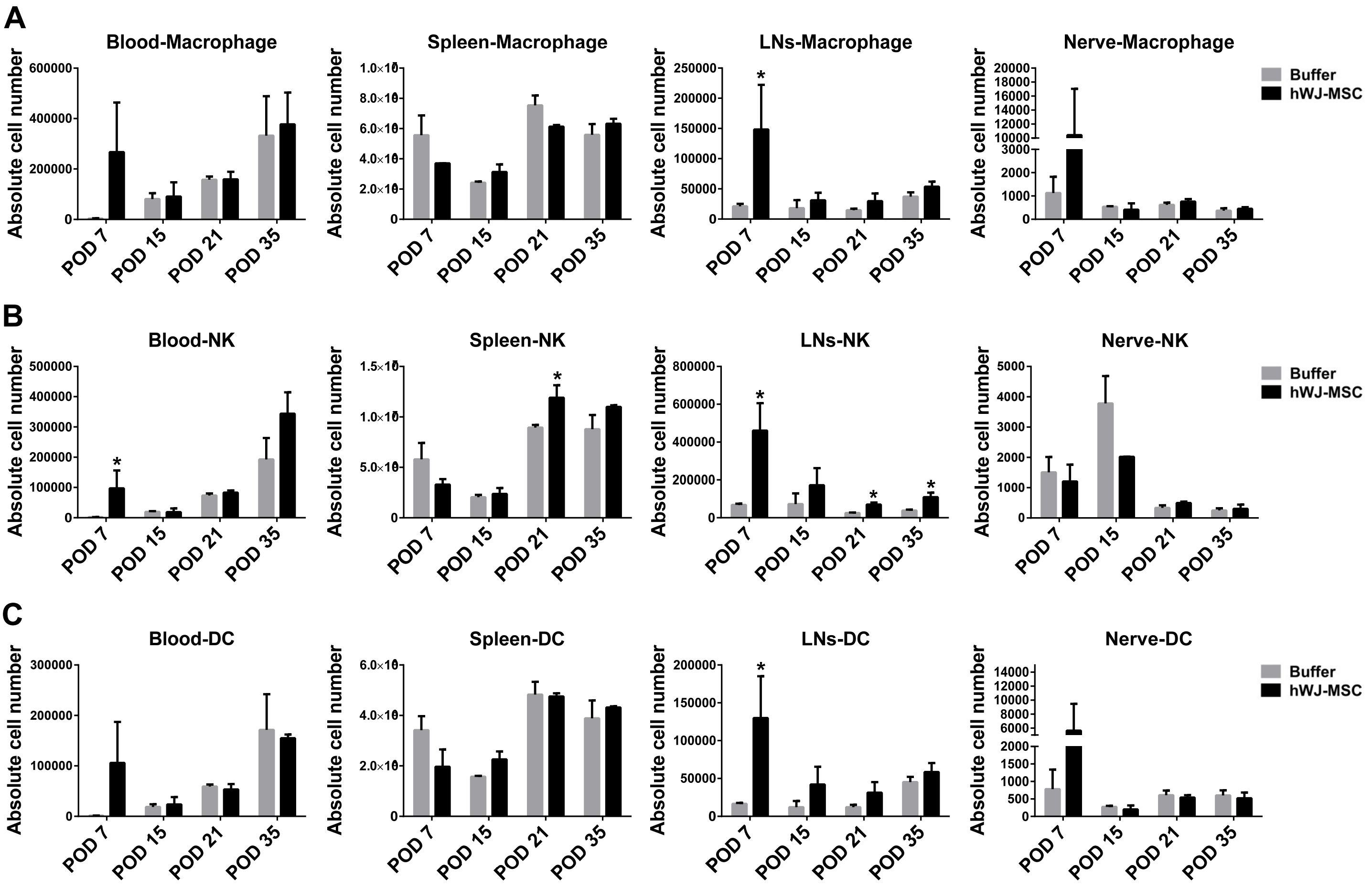

Figure 1. Effects of hWJ-MSC on innate immunity in sciatic nerve-injured mice. (A, B, C) The absolute number of macrophages, NKs, and DCs in blood, spleen, LNs, and nerve-infiltrating cells. Immune cells in the two groups (N=3) were harvested and analyzed using flow cytometry on POD7, 15, 21, and 35. The cells were stained to identify CD11b^{+} macrophages, NK1.1^{+} NKs, and CD11c^{+} DCs. The statistical data were represented as the mean ± SD. *P < 0.05.

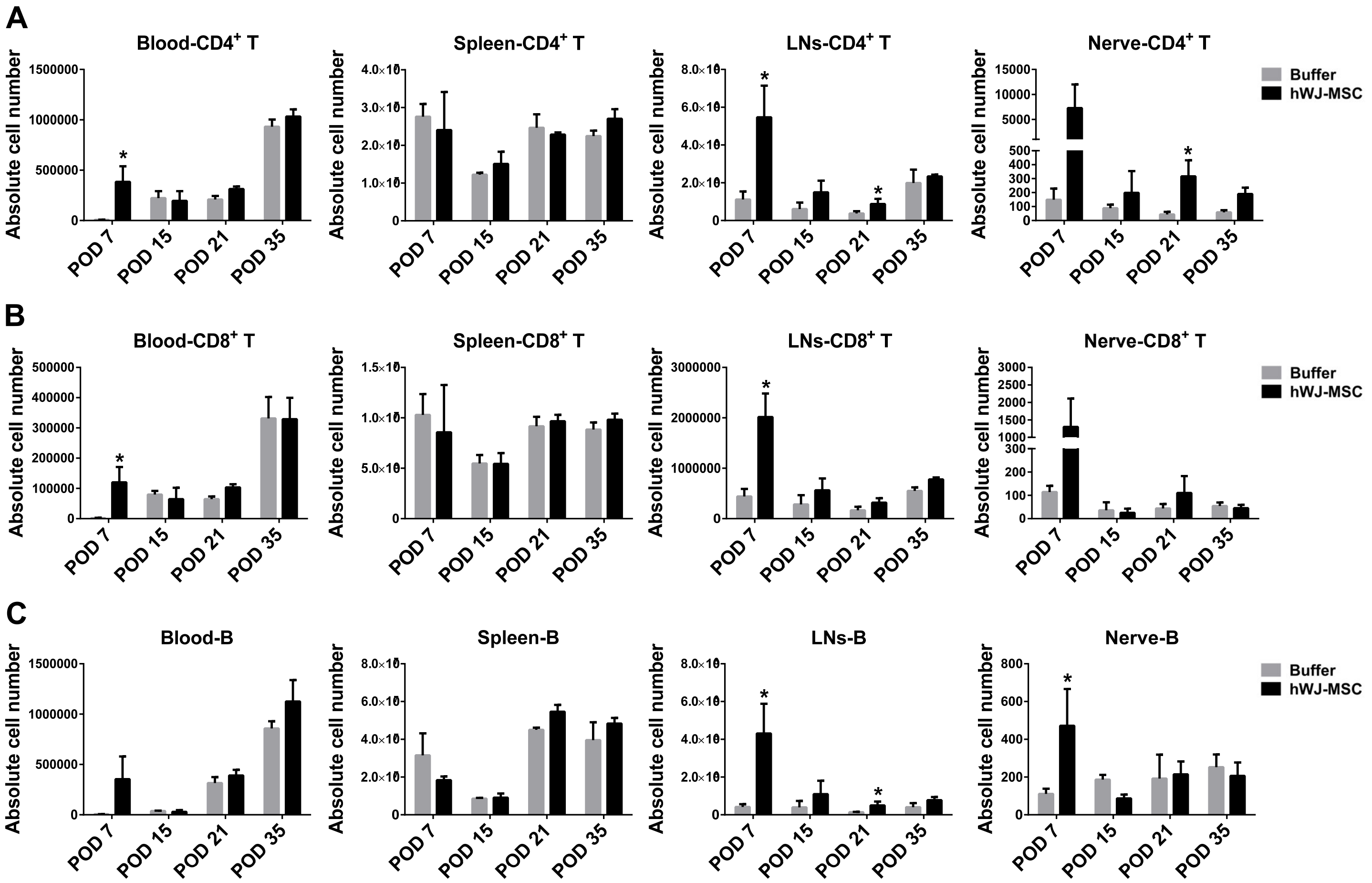

Figure 2. Effects of hWJ-MSC on adaptive immunity in sciatic nerve-injured mice. (A, B, C) The absolute number of $CD4^+$ T, $CD8^+$ T, and B cells in blood, spleen, LNs, and nerve-infiltrating cells. Immune cells in the two groups (N=3) were harvested and analyzed using flow cytometry on POD7, 15, 21, and 35. The cells were stained to identify $CD4^+$ T, $CD8^+$ T, and $CD19^+$ B cells. The statistical data were represented as the mean $\pm$ SD. *$P < 0.05$.

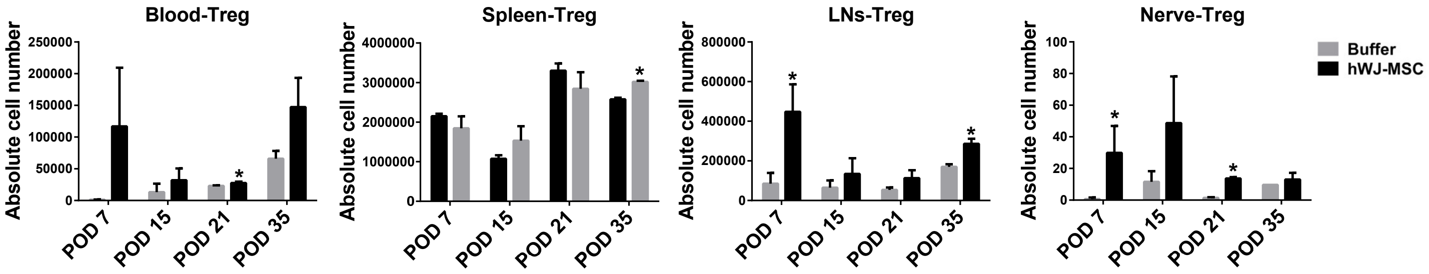

Figure 3. Effects of hWJ-MSC on Treg cells in sciatic nerve-injured mice. The absolute number of regulatory T cells (Tregs) in blood, spleen, LNs, and nerve-infiltrating cells. Tregs in the two groups (N=3) were harvested and analyzed using flow cytometry on POD7, 15, 21, and 35. The cells were stained to identify $CD4^+CD25^+FoxP3^+$ Tregs. The statistical data were represented as a mean ± SD. *P < 0.05.

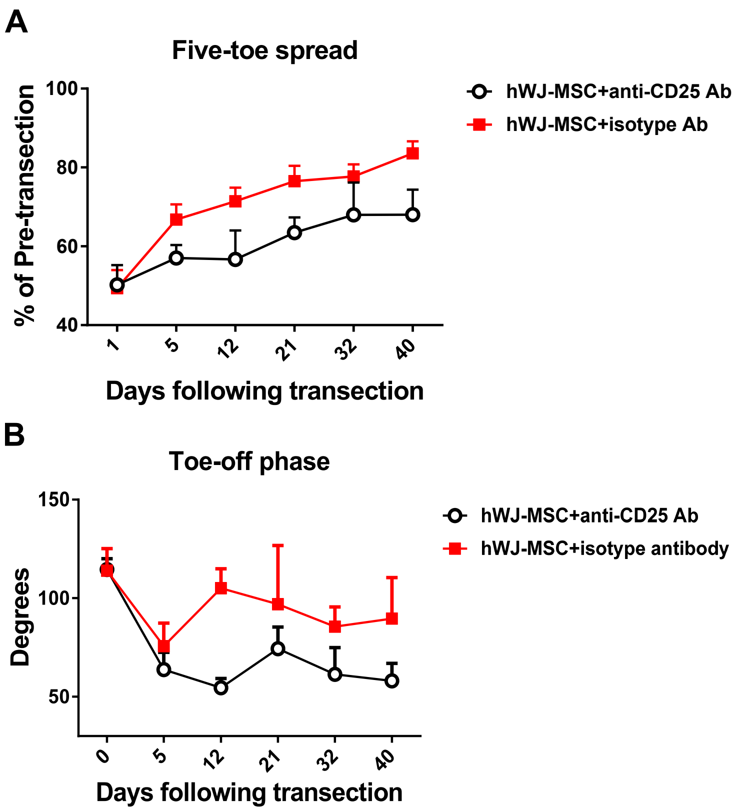

Figure 4. Anti-CD25 antibody administration eliminated the hWJ-MSC-mediated functional recovery in hindlimbs. (A) An anti-CD25 antibody worsened the functional recovery of the five-toe spread reflex. hWJ-MSC (5×10^5) in 50 μl were added to the space created in the musculature of each mouse. Progressive chart of the percentage of the pre-transection five-toe spread distance measured over time after the division of the sciatic nerve in the anti-CD25- and isotype-treated groups. The differences between the mean ± SD of the two groups were significant (P value < 0.05, one-way ANOVA; anti-CD25 vs isotype control, P = 0.01; Tukey's test). The average five-toe spread in the normal mouse group was 9.811 ± 0.057 mm. (B) The anti-CD25 antibody worsened gait stance and movement. The angles were measured in both groups after transection surgery. The mean ± SD values were significantly different in the two groups (P value < 0.05, one-way ANOVA; anti-CD25 vs isotype control, P = 0.04; Tukey's test). The average angle of the normal mouse group was 110.0° ± 0.72°.

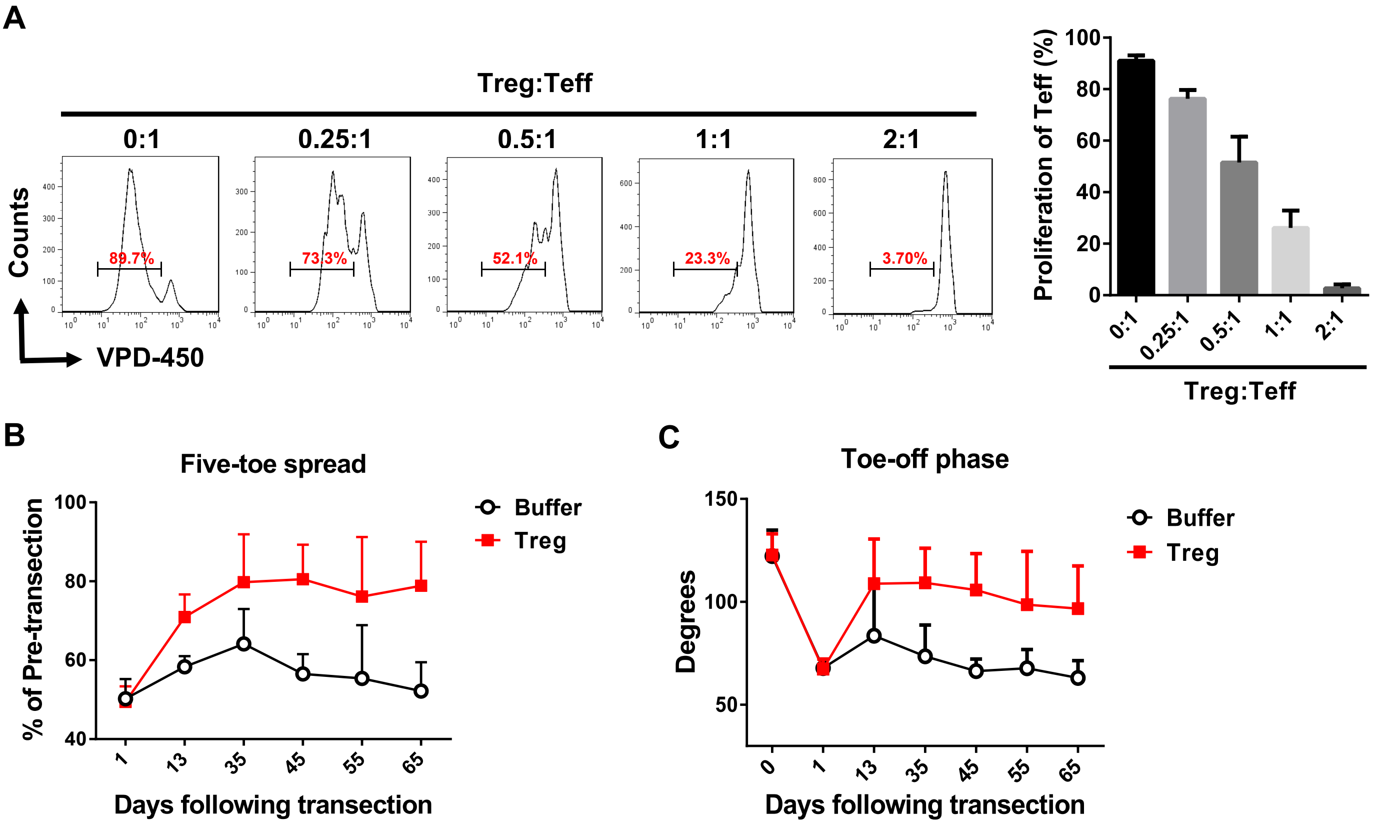

Figure 5. Treg administration improved the functional recovery of the sciatic nerve. (A) $CD4^+CD25^+$ Tregs (Treg) were isolated with a purity of >90% from naïve mice using a mouse $CD4^+CD25^+$ regulatory T cell isolation kit and an autoMACS instrument. $CD4^+CD25^-$ effector T cells (Teff) were isolated with a purity of >90% using an autoMACS instrument. Tregs were co-cultured with VPD-450-labeled effector T cells under anti-CD3 (2 μg/ml) and anti-CD28 (2 μg/ml) stimulation for 3 days before analysis using flow cytometry. The difference between the mean ± SD values of the Treg mixture (0.25~2:1) and Teff (0:1) groups was significant in all cases ($P < 0.005$, Student's t test). (B) Tregs promoted the functional recovery of the five-toe spread reflex. Isolated Tregs ($5x10^5$) with a purity of >90% were added to the space created in the musculature of each mouse. Progressive chart of the percentage of the pre-transection five-toe spread distance measured over time after the division of the sciatic nerve in the buffer and Treg groups. The differences between the mean ± SD values of the two groups were significant (P value < 0.05, one-way ANOVA; Treg vs buffer control, $P = 0.02$; Tukey's test). The average five-toe spread in the normal mouse group was 9.950 ± 0.158 mm. (C) Tregs promoted gait-stance and movement improvement. The angles were measured in both groups after the transection surgery. Comparison of the mean ± SD values in the two groups revealed the presence of a significant difference (P value < 0.005, one-way ANOVA; Treg vs buffer control, $P = 0.01$; Tukey's test). The average angle of the normal mouse group was 122.5° ± 2.89°.

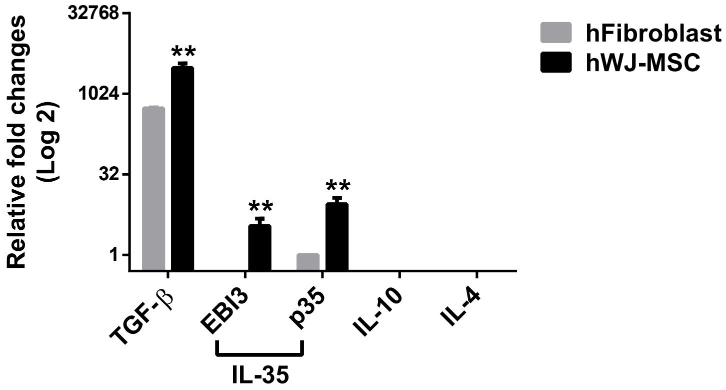

Figure 6. Quantification of hWJ-MSC-derived cytokine expression. The expression of mRNAs for Treg-associated cytokines, such as TGF-β, IL-10, IL-4, and IL-35 (which are composed of EBI3 and p35), in hWJ-MSC and fibroblasts was quantified using qPCR. The levels of expression of cytokines were was normalized to that of GAPDH. The difference between the mean ± SD values of the hWJ-MSC and fibroblast groups was significant ($P < 0.005$, Student's t test).

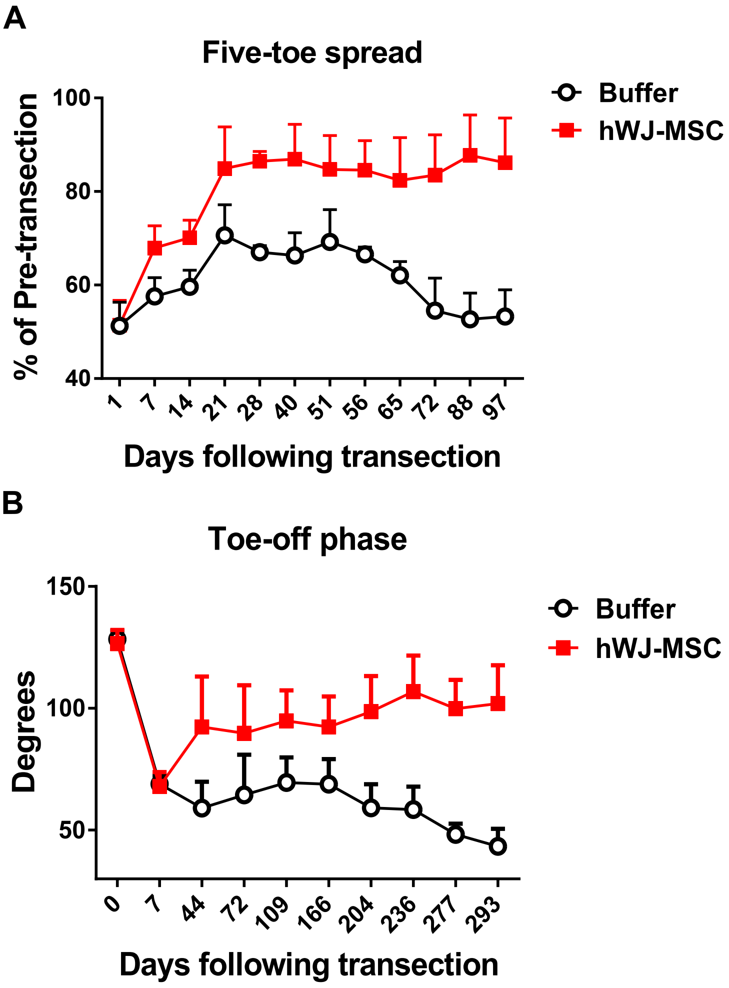

Supplementary Figure 1. hWJ-MSC promoted the functional recovery of injured and repaired sciatic nerves in the extremities. (A) hWJ-MSC accelerated the functional recovery of the five-toe spread reflex. Progressive chart of the percentage of the pre-transection five-toe spread distance measured over time after the division of the sciatic nerve in the buffer and hWJ-MSC groups. The differences between the mean ± SD of the buffer and hWJ-MSC groups were significant (*P* value < 0.0001, one-way ANOVA; buffer vs hWJ-MSC, *P* = 0.0001; Tukey's test). The average five-toe spread in the normal mouse group was 9.911 ± 0.04832 mm. (B) hWJ-MSC restored normal gait stance and movement. The angles were measured in the buffer and hWJ-MSC groups for up to 293 days after the transection surgery. Comparison of the mean ± SD values in the two groups revealed the presence of a significant difference (*P* = 0.0008, one-way ANOVA; buffer vs hWJ-MSC, *P* = 0.0030; Tukey's test). The average angle of the normal mouse group was 128.0° ± 0.8376°.

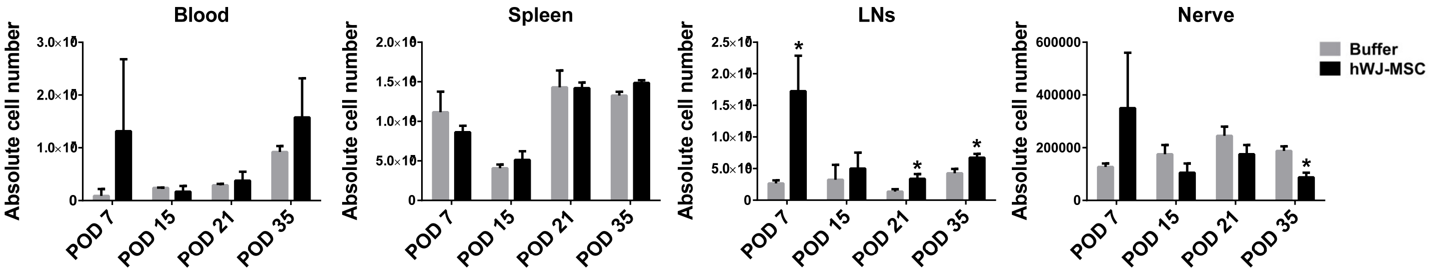

Supplementary Figure 2. Total absolute number of immune cells in blood, spleen, LNs, and nerve-infiltrating cells between the buffer and hWJ-MSC groups over time. Immune cells in the two groups (N=3) were harvested and analyzed using flow cytometry on POD7, 15, 21, and 35. The statistical data were represented as the mean ± SD. *P < 0.05.

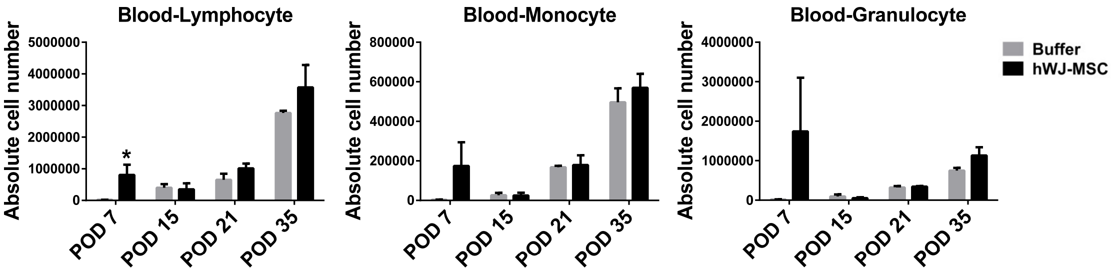

Supplementary Figure 3. The absolute number of lymphocytes, monocytes, and granulocytes in blood between the buffer and hWJ-MSC groups over time. Blood cells in the two groups (N=3) were harvested and analysed using flow cytometry on POD7, 15, 21, and 35. The statistical data were represented as the mean ± SD. *P < 0.05.

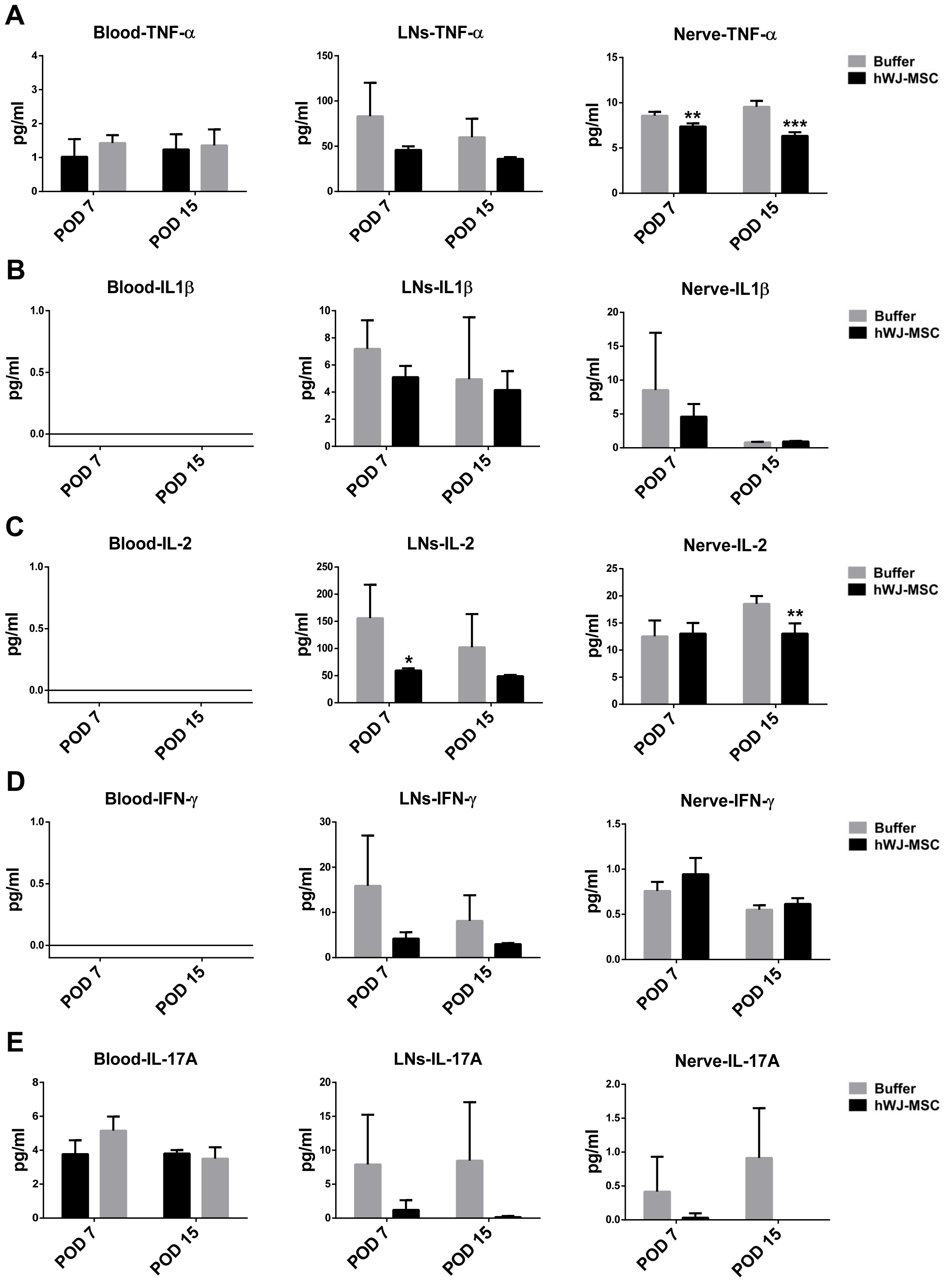

Supplementary Figure 4. Pro-inflammatory cytokine expression. Blood serum, LNs, and anastomosed sciatic nerves in the buffer and hWJ-MSC groups (N=3) were harvested on POD7 and 15. The cytokine expression of TNF-α, IL1β, IL-2, IFN-γ, and IL17A was analyzed using ProcartaPlex Immunoassays and Luminex instrument platform. The statistical data were represented as the mean ± SD. *P < 0.05.

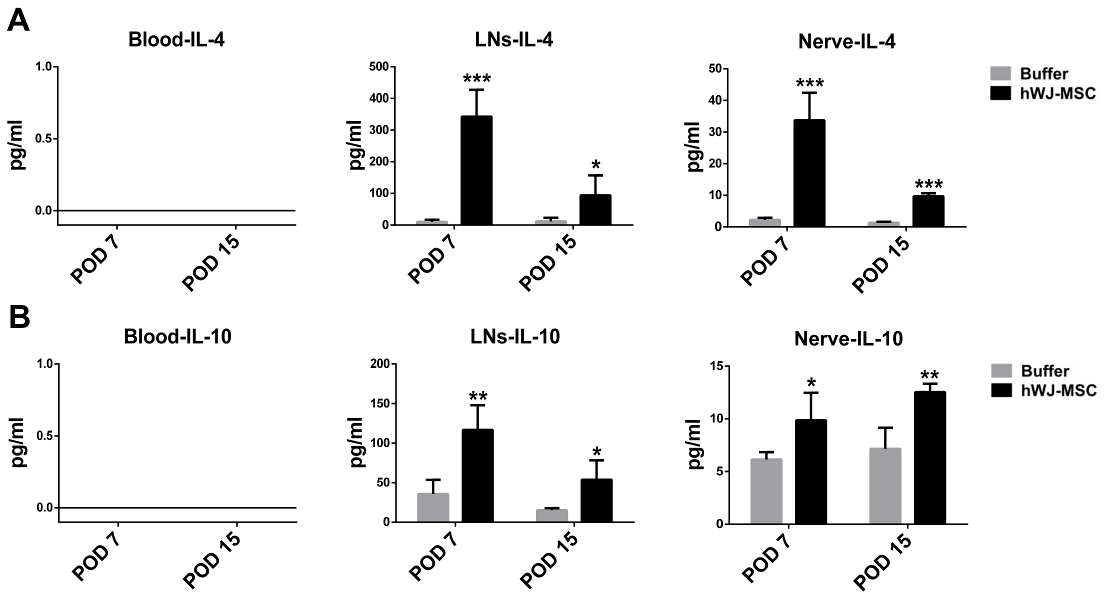

Supplementary Figure 5. Anti-inflammatory cytokine expression. Blood serum, LNs, and anastomosed sciatic nerves in the buffer and hWJ-MSC groups (N=3) were harvested on POD7 and 15. The cytokine expression of IL-4 and IL-10 was analyzed using ProcartaPlex Immunoassays and Luminex instrument platform. The statistical data were represented as the mean ± SD. *P < 0.05.

Blood-Treg	Buffer	hWJ-MSC
POD 7	1224 ± 233.4	117061 ± 53390
POD 15	13330 ± 7642	31959 ± 10710
POD 21	23112 ± 446.7	27566 ± 1261
POD 35	55985 ± 18662	147374 ± 26620

Spleen-Treg	Buffer	hWJ-MSC
POD 7	2151000 ± 37634	1844000 ± 175099
POD 15	1074000 ± 53108	1533000 ± 210611
POD 21	3303000 ± 104629	2845000 ± 242526
POD 35	2580000 ± 26508	3020000 ± 17068

LNs-Treg	Buffer	hWJ-MSC
POD 7	84991 ± 31172	447631 ± 80008
POD 15	65129 ± 20588	134008 ± 45827
POD 21	53685 ± 6745	113051 ± 22955
POD 35	169513 ± 9513	286464 ± 18002

Nerve-Treg	Buffer	hWJ-MSC
POD 7	0.6482 ± 0.5526	29.91 ± 9.781
POD 15	11.56 ± 3.889	48.71 ± 17.02
POD 21	1.333 ± 0.3333	13.73 ± 0.4807
POD 35	8.33 ± 1.33	13.03 ± 2.421

Supplementary Figure 6. The absolute number (mean ± SD) of Tregs in blood, spleen, LNs, and nerve-infiltrating cells over time between the buffer and hWJ-MSC groups.
